# Differential alternative splicing genes and isoform co-expression networks of *Brassica napus* under multiple abiotic stresses

**DOI:** 10.3389/fpls.2022.1009998

**Published:** 2022-10-13

**Authors:** Lingli Yang, Li Yang, Chuanji Zhao, Jie Liu, Chaobo Tong, Yuanyuan Zhang, Xiaohui Cheng, Huifang Jiang, Jinxiong Shen, Meili Xie, Shengyi Liu

**Affiliations:** ^1^ National Key Laboratory of Crop Genetic Improvement, College of Plant Science and Technology, Huazhong Agricultural University, Wuhan, China; ^2^ The Key Laboratory of Biology and Genetic Improvement of Oil Crops, The Ministry of Agriculture and Rural Affairs, Oil Crops Research Institute of the Chinese Academy of Agricultural Sciences, Wuhan, China; ^3^ Biosystematics Group, Wageningen University and Research, Wageningen, Netherlands

**Keywords:** *Brassica napus*, abiotic stress, RNA-seq, alternative splicing, WGCNA, RBPs, TFs

## Abstract

Alternative splicing (AS) is an important regulatory process that affects plant development and stress responses by greatly increasing the complexity of transcriptome and proteome. To understand how the AS landscape of *B. napus* changes in response to abiotic stresses, we investigated 26 RNA-seq libraries, including control and treatments with cold, dehydration, salt, and abscisic acid (ABA) at two different time points, to perform comparative alternative splicing analysis. Apparently, AS events increased under all stresses except dehydration for 1 h, and intron retention was the most common AS mode. In addition, a total of 357 differential alternative splicing (DAS) genes were identified under four abiotic stresses, among which 81 DAS genes existed in at least two stresses, and 276 DAS genes were presented under only one stress. A weighted gene co-expression network analysis (WGCNA) based on the splicing isoforms, rather than the genes, pinpointed out 23 co-expression modules associated with different abiotic stresses. Among them, a number of significant hub genes were also found to be DAS genes, which encode key isoforms involved in responses to single stress or multiple stresses, including RNA-binding proteins, transcription factors, and other important genes, such as *RBP45C*, *LHY*, *MYB59*, *SCL30A*, *RS40*, *MAJ23.10*, and *DWF4*. The splicing isoforms of candidate genes identified in this study could be a valuable resource for improving tolerance of *B. napus* against multiple abiotic stresses.

## 1 Introduction

Alternative splicing (AS) is a common phenomenon in eukaryotes, which can use different splicing sites to produce multiple mRNA isoforms from the same pre-mRNA, thus increasing transcriptomic and proteomic diversity (Reddy et al., 2013; Staiger and Brown, 2013). More than 95% of intron-containing genes undergo AS in humans (Wang et al., 2008), and the proportion is as high as 70% in plants (Shen et al., 2014b; Thatcher et al., 2014; Zhang et al., 2017; Ruan et al., 2018). The selection of alternative splicing sites is determined by both *cis*-regulatory elements and *trans*-splicing factors. The *cis*-regulatory elements contain splice sites, polypyrimidine tract, branch point, and exonic and intronic splicing enhancer/silencer, which are the binding sites of splicing factors. *Trans*-splicing factors control the selection of splice sites and instruct splice assembly, including serine-/arginine-rich (SR) proteins and heterogeneous nuclear ribonucleoproteins (hnRNPs), in which SR proteins promote splicing, while hnRNPs do the opposite, both belonging to the important RNA-binding proteins (Syed et al., 2012; Erkelenz et al., 2013; Geuens et al., 2016). Splicing factors are widely alternatively spliced in plant development and responses to abiotic stresses, thus affecting the AS patterns and expression of downstream target genes (Staiger and Brown, 2013; Thatcher et al., 2014; Gu et al., 2019).

There are five basic types of AS events, namely, exon skipping (ES), intron retention (IR), alternative 5′ splice sites (A5SS), alternative 3′ splice sites (A3SS), and mutually exclusive exons (MEs). The proportion of AS events varies in different species: IR is the most common AS event in plants, while ES is the most prevalent one in animals (Shen et al., 2014b). AS can augment proteome diversity by generating diverse protein isoforms (Syed et al., 2012; Kruse et al., 2020), which is also coupled to mRNA stability and translation through nonsense-mediated decay (NMD) and microRNAs (miRNAs) regulation (Reddy et al., 2013; Chen et al., 2018). It was reported that 35% of AS events in polysome-associated mRNAs were translated into proteins, most of which resulted in diverse proteins in *Arabidopsis* (Yu et al., 2016). About 11%–18% of intron-containing genes were alternatively spliced to generate transcripts containing premature termination codons (PTCs) and subsequently coupled with the NMD pathway in *Arabidopsis*, such as *CCA1*, *SR34a*, *GRP8*, and *GRP7* (Kalyna et al., 2012; Drechsel et al., 2013). Similarly, 32% of AS genes were subjected to NMD in *Physcomitrella patens* (Lloyd et al., 2018). In addition, a slice of transcripts produced by AS could obtain or lose miRNA binding sites, thus varying their expression by the regulation of miRNA (Iwakawa and Tomari, 2015; Chen et al., 2018).

AS is an important regulatory process for plants to respond to various stresses (Laloum et al., 2018). It has been proven that many vital genes, including those encoding transcription factors (TFs) and RNA-binding proteins (RBPs), underwent alternative splicing due to environmental stresses. For instance, in *Arabidopsis*, the transcription factor *IDD14* produced a truncated protein variant lacking a functional DNA binding domain through intron retention, which was unable to activate the expression of starch-degrading enzymes, thereby resulting in starch accumulation under low temperatures (Seo et al., 2011). In *Arabidopsis* and rice, the heat shock transcription factor *HsfA2* produced *HsfA2-III* isoform, instead of the PTC-containing *HsfA2-II* isoform, leading to the generation of truncated proteins under heat stress (Sugio et al., 2009; Liu et al., 2013; Cheng et al., 2015). Another crucial abiotic stress regulator, *DREB2B*, was also reported to be alternatively spliced in wheat and rice. Under drought and salt stresses, wheat *WDREB2* was prone to produce *WDREB2α* and *WDREB2γ* isoforms, rather than *WDREB2β* encoding a non-functional polypeptide (Terashima and Takumi, 2009). Similarly, the functional isoform *OsDREB2B2*, but not the PTC-containing non-functional isoform *OsDREB2B1*, was generated to enhance stress tolerance in rice under high temperature and drought stresses (Matsukura et al., 2010), highlighting the conservation of AS regulation in plants. As to RBPs, multiple studies have also focused on the splicing pattern in response to stresses. For example, one SR gene, *atSR30*, could generate five different isoforms *atSR30* mRNA1–5 *via* AS, while it tended to produce atSR30 mRNA1 and atSR30 mRNA4 under high-light irradiation and atSR30 mRNA1 with the treatment of paraquat in *Arabidopsis* (Tanabe et al., 2007). In addition, other SR genes, such as *SR34*, *SR34b*, *RS31*, *RS40*, *RSZ32*, and *SR33*/*SCL33*, in *Arabidopsis*, showed prominent changes in AS patterns under various abiotic treatments, including hormones and cold or heat stresses (Palusa et al., 2007). For example, *SR45*, *SR30*, and *SR34*, along with nuclear ribonucleic protein U1A, were more likely to produce AS events under high temperatures in grape (Jiang et al., 2017). A splicing factor *RBM25* underwent AS activated by ABA, which was responsible for splicing many pre-mRNAs participated in ABA signal transduction pathways in *Arabidopsis* (Zhan et al., 2015; Cheng et al., 2017). Meanwhile, other types of genes were also reported recently. For example, the cyclin-D3-1-like gene may respond to environmental stresses *via* alternative splicing in *Brassica napus* (Guo et al., 2019), and the gene encoding cyclophilin 38 was induced to increase IR events nearly 30-fold under heat stress in *P. patens* (Chang et al., 2014). In the past, the AS events of most genes in response to stresses were identified by wet-lab experiments, while recent studies have developed the methodology for genome-wide identification of AS events and AS networks by transcriptomics, so that more splicing variations can be detected. For example, the AS landscapes ascribable to individual stress such as *Sclerotinia sclerotiorum*, *Leptosphaeria maculans*, and boron deficiency in *B. napus* have been illustrated (Gu et al., 2019; Ma et al., 2019; Ma et al., 2020). The AS dynamics of *Arabidopsis*, maize, and wheat, caused by salt, drought, and cold and heat stresses, alone or in combination, were also elaborated in detail (Ding et al., 2014; Thatcher et al., 2016; Liu et al., 2018).

Global agriculture is confronted with tremendous environmental changes, such as extreme temperatures, drought, and salt stresses, which are critical limiting factors influencing the growth and yield of crops such as rapeseed (*Brassica napus L.*), the worldwide second largest oilseed crop. Therefore, identifying genes with broad-spectrum effects on different stress responses is one of the long-term goals of genetic improvement in rapeseed. With the development of sequencing technology, many mature research methods have been widely used to identify potential genes involved in response to abiotic stresses, including genome-wide association studies (Zhang et al., 2015; Liu et al., 2021; Wassan et al., 2021) and comparative transcriptome analyses that focus primarily on differential expression analyses (Du et al., 2016; Pu et al., 2019; Raza et al., 2021). Nevertheless, these methodologies rarely consider the widespread AS events in plants, which, as described above, play an important role in response and adaptation to various abiotic stresses. Hence, identifying genes that alter AS modes in response to stresses could also provide resources for improving stress tolerance. However, comprehensive analysis of AS responsive to multiple abiotic stresses such as heat, drought, salt, and ABA in *B. napus* is still rarely available.

In this study, to identify candidate resistance genes in response to multiple abiotic stresses, comparative differential AS analyses were performed between control and treatments subjected to cold, dehydration, salt, and ABA at two different time points in *B. napus*. Weighted gene co-expression network analysis (WGCNA) was implemented to determine the regulatory network of *B. napus* in reaction to different abiotic stresses based on isoform expression level. This research described the global dynamics of AS isoforms, which would deepen our understanding of the response of *B. napus* to various abiotic stresses. In addition, the findings of the candidate stress responsive genes could assist in rapeseed variety breeding with extensive abiotic stress tolerance.

## 2 Materials and methods

### 2.1 RNA-seq data download, reads alignment, and transcript assembly

The RNA-seq data of *B. napus* leaves before and after cold, dehydration, salt, and ABA treatments at two different time points were downloaded from the National Genomics Data Center with project number CRA001775 (https://bigd.big.ac.cn/), of which data for replicate 1 of 1 h of dehydration was removed from subsequent analysis due to poor biological reproducibility (Zhang et al., 2019). Raw reads were first filtered by removing reads containing adapter, ploy-N, and low-quality reads using Trimmomatic v0.36 (Bolger et al., 2014). The generated paired-end clean reads were then aligned to the reference genome using Hisat2 v2.2.0 (Chalhoub et al., 2014; Kim et al., 2015). Furthermore, the in-house perl scripts were used to exclude reads aligned to multiple chromosome positions to minimize pseudomorphisms caused by ambiguous mapping. Finally, StringTie v2.1.5 was used to construct and identify both known and novel transcripts at the base of alignments and to quantify transcripts per million (TPM) of each isoform across all samples (Pertea et al., 2015).

### 2.2 AS landscape and differential alternative splicing analysis

Based on the gtf annotations generated by StringTie, Astanavista v4.0 was adopted to analyze the AS patterns of each sample (Foissac and Sammeth, 2007), including intron retention, exon skipping, alternative acceptors, and alternative donors events. Differential alternative splicing (DAS) genes are defined as genes that undergo differential AS events and produce different types of isoforms under different stress conditions compared with the control. DAS events between control and different treatments were respectively quantified using rMATS. rMATS is a software for differential alternative splicing analysis from RNA-Seq data with biological replicates. rMATS uses likelihood-ratio test to calculate the p-value and false discovery rate (FDR) to denote the difference in inclusion level (IncLevel, also designated as ψ) between the two groups of samples. First, the IncLevel1 and IncLevel2 from the two groups of samples were calculated, respectively, as follows: ψ = (I/LI)/(I/LI + S/LS). For example, ψ = Inclusion Level (IncLevel1), where I = number of reads mapped to the exon inclusion isoform (IC_SAMPLE_1), S = number of reads mapped to the exon skipping isoform (SC_SAMPLE_1), LI = effective length of the exon inclusion isoform (IncFormLen), and LS = effective length of the exon skipping isoform (SkipFormLen). Each biological replicate for one group had an IncLevel value, and IncLevelDifference **(**ΔIncLevel**) =** average(IncLevel1) − average(IncLevel2). rMATS uses a likelihood-ratio test to calculate the p-value for the difference in the mean IncLevel values between two groups of samples. Benjamini–Hochberg method (FDR) was used to correct p-value, and the thresholds of |ΔIncLevel| ≥ 0.1 and FDR < 0.05 were used to identify DAS. The command “python rmats.py –b1 control.txt –b2 treatment.txt -t paired –readLength 144 –gtf stringtie_merge.gtf –od treatment.dir –nthread 20 –cstat 0.1 –libType fr-unstranded” was used for this process (Shen et al., 2014a). Moreover, rmats2sashimiplot was used to visualize DAS events (https://github.com/Xinglab/rmats2sashimiplot).

### 2.3 Weighted gene co-expression network analysis of isoforms

In order to investigate the regulatory relationships between isoforms, WGCNA was performed on the top 50% transcripts selected based on their TPM variances using R v3.6.2. This analysis consisted of a series of steps, including sample clustering, outlier detection, soft threshold selection, one-step network construction, module identification, and module-treatment relationship analysis (Langfelder and Horvath, 2008). Subsequently, in-house perl scripts were used to filter out pair-wise co-expressed transcripts with weighted value <0.15 to obtain significant co-expression networks, which were finally visualized in Cytoscape v3.8.2 (Kohl et al., 2011). Isoforms whose absolute value of kME (eigengene connectivity) was not <0.8 (|kME|≥0.8) were designated as hub isoforms within each module. The average TPM values of different biological replicates were converted to log_2_ (TPM+1) for isoform heatmap visualization using R package pheatmap.

### 2.4 Functional annotation and gene ontology enrichment analysis

To gain functional annotation of the differentially spliced genes in *B. napus*, all genes were aligned against *Arabidopsis* protein database using local Blast similarity search, and the best hits with e‐value <1e−10 were retained. The functional information of *Arabidopsis* genes was extracted from TAIR database (http://www.arabidopsis.org/). Moreover, Gene Ontology (GO) enrichment analyses were performed using software TBtools (Chen et al., 2020).

## 3 Results

### 3.1 AS landscape of *B. napus* in response to different abiotic stresses

To identify the AS landscape of *B. napus* in response to multiple abiotic stresses, RNA-seq data of 26 samples from leaves of cultivar Zhongshuang 11 (ZS11) were downloaded from NGDC database to perform comparative alternative splicing analyses. These samples included control and treatments with 1 and 8 h of dehydration, 4 and 24 h of cold, NaCl and ABA (Zhang et al., 2019). The downloaded data were then subjected to quality trimming, reads mapping, transcript assembly, followed by transcript merging to quantify TPM for each isoform (**Supplementary Table 1**). These analyses resulted into a total of 28,720 new transcripts across all these conditions (**Supplementary Table 2**).

**Figure 1:**
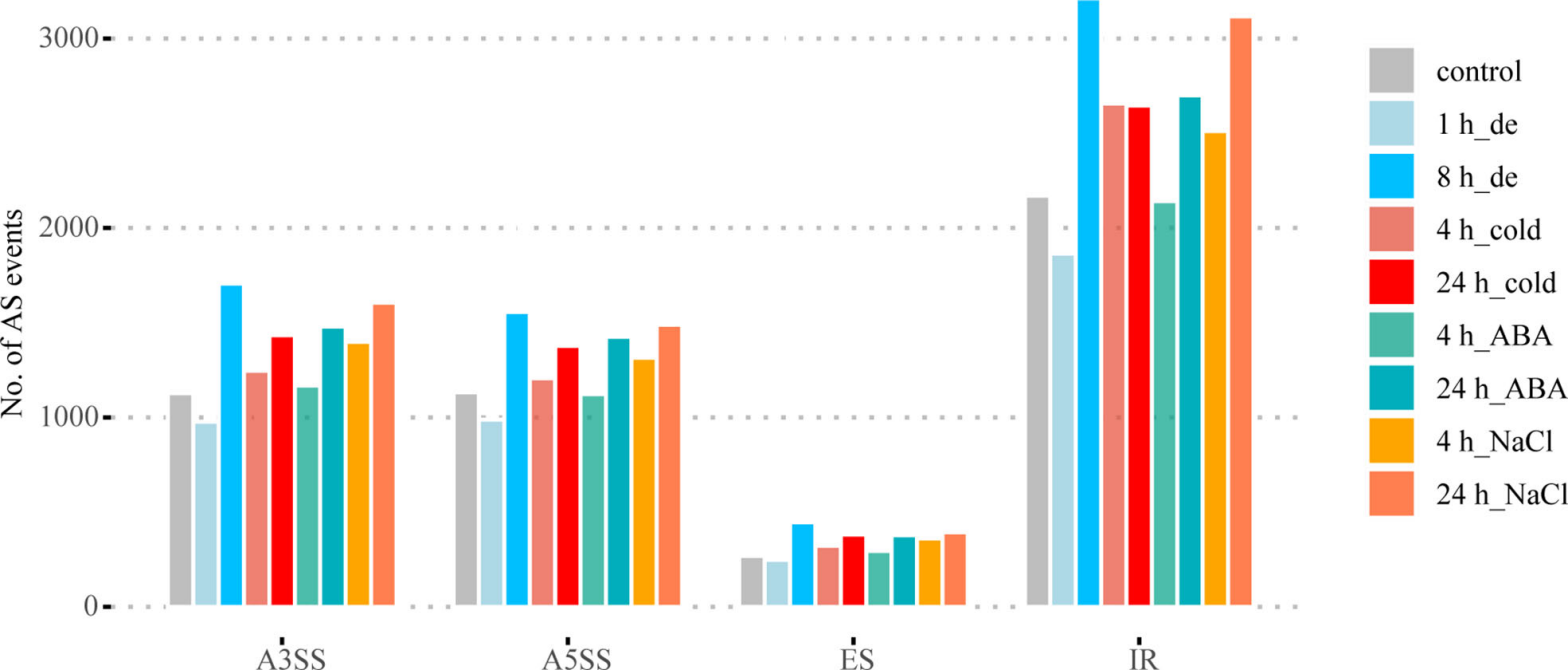
AS landscapes of control and treatments with dehydration, cold, ABA, and NaCl at two different time points in *B. napus.* A3SS, alternative 3′ splice site; A5SS, alternative 5′ splice site; ES, exon skip; IR, intron retain.

The frequency of four different types of AS events was identified in *B. napus* under different abiotic stress conditions using Astalavista v4.0 (Foissac and Sammeth, 2007). As shown in **Figure 1** and **Supplementary Table 1**, IR was the most common among the four AS events, accounting for 45.94% of all AS events identified under different stress conditions on average, while ES events accounted for the lowest proportion, with an average of just 5.67%. The average ratios of A5SS and A3SS events were very similar, 23.96% and 23.88%, respectively. These results were consistent with the frequency distribution of alternative splicing in plant genomes reported in previous studies (Zhang et al., 2017; Ruan et al., 2018). In general, the AS events of most treatments increased with the extension of stress treatment time compared with the control (**Figure 1**).

### 3.2 AS transcriptional dynamics in *B. napus* after stress treatment

**Figure 2:**
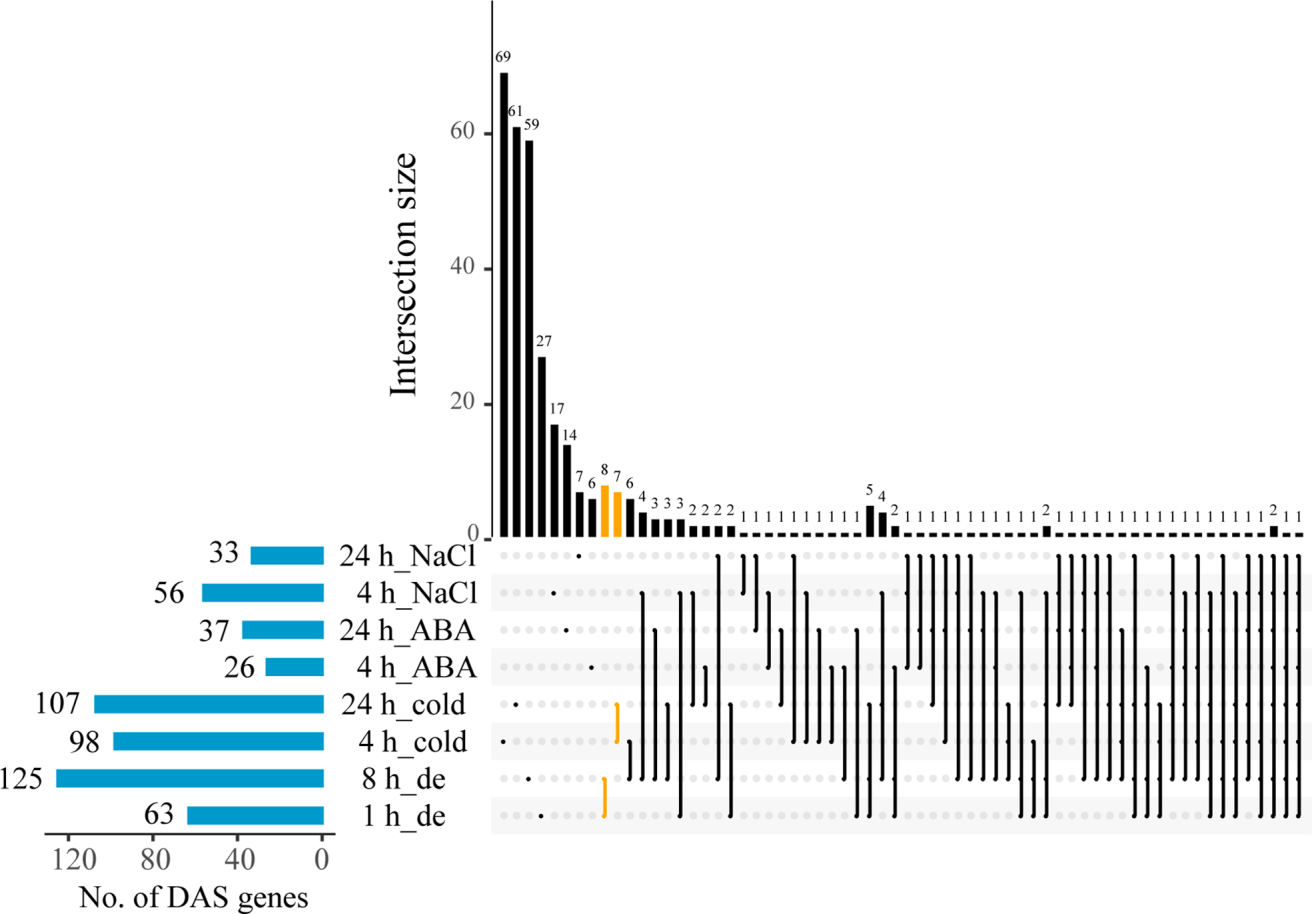
The numbers of differentially alternative splicing (DAS) genes between control and treatments with dehydration, cold, ABA, and NaCl at two different time points in *B*. *napus*.

In order to study the AS transcriptional dynamics at different time points upon the treatments of four stresses, DAS genes were identified and systematically investigated between control and each of the treatments. Overall, this analysis resulted into a total of 357 DAS genes by integrating all comparative data sets. More DAS genes under treatments of dehydration and cold were observed than that of ABA and NaCl stresses, implying a stronger response of AS evoked by the former in *B. napus* (**Figure 2**). Specifically, the number of DAS genes was significantly variable across different treatments. A striking increase in the number of DAS genes (63 to 125) was observed with the time course of dehydration stress treatment, while the number of DAS genes enhanced slightly under ABA and cold stresses, from 26 to 37 and from 98 to 107 at 4 and 24 h, respectively (**Figure 2**). However, with the treatment course of NaCl stress, the number of DAS genes was found to show a moderate reduction from 56 to 33 (**Figure 2**). Comparative analysis of DAS genes between different stress treatments revealed that 81 DAS genes were detected under at least two stresses (**Supplementary Table 3**). For example, exon skipping of *MSTRG.1012* was identified between control and several stresses including ABA, dehydration, and NaCl (**Supplementary Figure 1A**). In addition, the number of DAS genes specifically induced by individual stress was largest under cold stress (137), followed by dehydration (94), NaCl (25), and ABA (20) (**Supplementary Table 3**). For example, differential ES event of *MSTRG.17416* was identified only at 8 h of dehydration compared with control (**Supplementary Figure 1B**). Overall, these results implied that ~23% of DAS genes could be induced by multiple stresses, and most DAS genes were independently induced by individual stress.

In total, 286 homologs of 357 *B. napus* DAS genes were identified in *A. thaliana* (**Supplementary Table 3**), of which 45 (corresponding to 53 DAS genes in *B. napus*) were reported to be associated with abiotic stress (Naika et al., 2013). Moreover, 125 out of the 286 homologous genes have been previously shown to be AS genes according to the ASIP database (Wang and Brendel, 2006). These DAS genes included RBPs, TFs, and other important genes, such as *RBP45C*, *LHY*, *MYB59*, *SCL30A*, *RS40*, *MAJ23.10*, and *DWF4*. By GO enrichment analysis, all 357 DAS genes were found to be mainly involved in splicing and metabolic processes, including mRNA metabolism, mRNA splicing *via* spliceosome, RNA splicing *via* transesterification reactions, photoperiodism, and regulation of transcription (**Supplementary Table 4**).

### 3.3 Co-expression network analysis and identification of hub DAS genes in response to abiotic stresses

**Figure 3:**
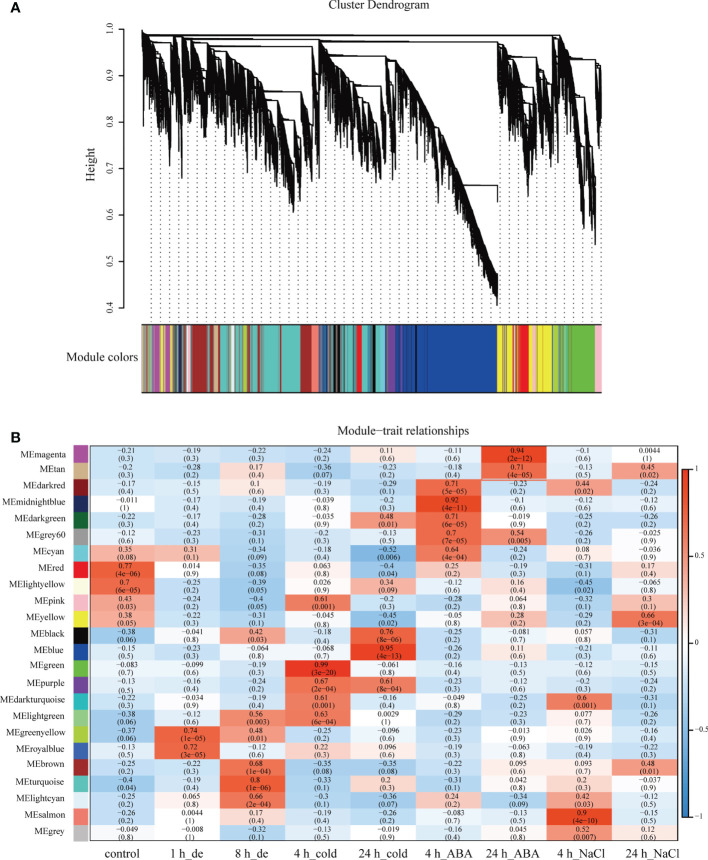
Co-expression network generation on isoform level by WGCNA. **(A)** Hierarchical cluster tree showing co-expression modules identified by WGCNA. Each leaf in the tree represents one isoform. The major tree branches constitute 23 modules, labeled with different colors. **(B)** Heatmap of correlation between modules and treatments. Each row corresponds to a module, labeled with a color as in panel **(A)**.

To explore the potential genes/isoforms in response to various stress treatments, WGCNA was performed at the base of 35,527 identified isoforms with high expression level and top 50% TPM variances, to generate isoform co-expression network. A scale-free network was constructed based on weight value *β* of 6, and 23 co-expression modules were obtained accordingly (**Figure 3**). The generated modules were color-coded, and of note was that the gray module contained isoforms not assigned to any other modules. Remarkably, the isoform numbers of different modules varied greatly, ranging from 85 to 8,241, among which the turquoise module possessed most isoforms (8,241), while the dark turquoise module had the least number of isoforms (85) (**Supplementary Figure 2**).

**Figure 4:**
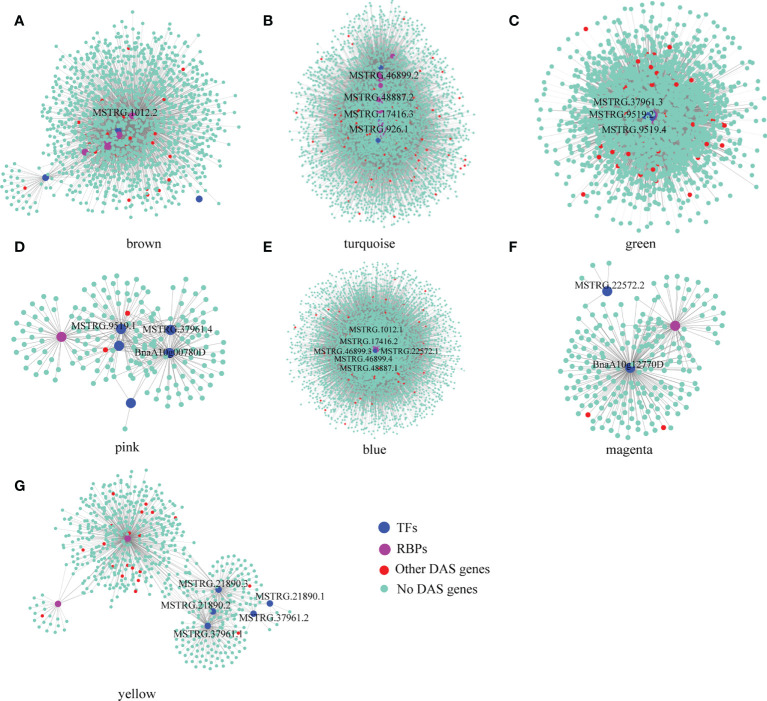
Visualization of the stress-related co-expression networks by Cytoscape. Panels **(A–G)**, respectively, represented brown, turquoise, green, pink, blue, magenta, and yellow module. Using Cytoscape, the purple nodes represent the isoforms of the RBPs of DAS genes, the blue nodes represent the isoforms of TFs of DAS genes, the red nodes represent the isoforms of other DAS genes, and the light blue nodes represent other connected isoforms.

Here, we identified 576 isoforms whose precursor genes were identified as 257 DAS genes, of which approximately 50% genes (131) produced hub isoforms in 16 stress-related modules, such as brown-8 h_de, turquoise-8 h_de, green-4 h_cold, pink-4 h_cold, blue-24 h_cold, magenta-24 h_ABA, and yellow-24 h_NaCl, suggesting that alternative splicing may play a central role in co-expression regulation of *B. napus* in response to abiotic stresses (**Figure 4** and **Supplementary Table 5**). To explore gene functions within stress-related modules, GO enrichment analysis was performed, and the top 20 significantly enriched terms are shown in **Supplementary Figure 3**. For genes belonging to brown and turquoise modules associated with 8 h of dehydration, the significantly enriched GO terms (p < 0.05) were various processes of carbohydrate metabolism, water-soluble vitamin biosynthetic process, coenzyme biosynthetic/metabolic process, ubiquitin-dependent protein catabolic process, and splicing-related processes such as RNA splicing, nucleosome assembly, and DNA conformation change. As for 4 h cold-stress-related pink and green modules, the genes were mainly involved in the molecule metabolism/biosynthesis, transport, and the regulation of these processes. With the extension of cold stress, the blue module was identified to be related, and various processes mediating proteins synthesis and transport were detected, such as translation, translational elongation, ribosome biogenesis, cellular protein metabolic process, and protein transport. Additionally, the magenta and yellow modules were found to be associated with the later stages of ABA and NaCl treatments, respectively. The genes were mainly enriched in ion transports, nucleotide metabolic process, and fatty acid biosynthetic process for the former, while the genes were mostly responsible for carboxylic acid, cellular amino acid, and small molecule metabolic/biosynthetic/catabolic processes for the latter.

#### 3.3.1 Hub DAS genes in response to single abiotic stress

##### 3.3.1.1 Hub DAS genes under dehydration stress

**Figure 5:**
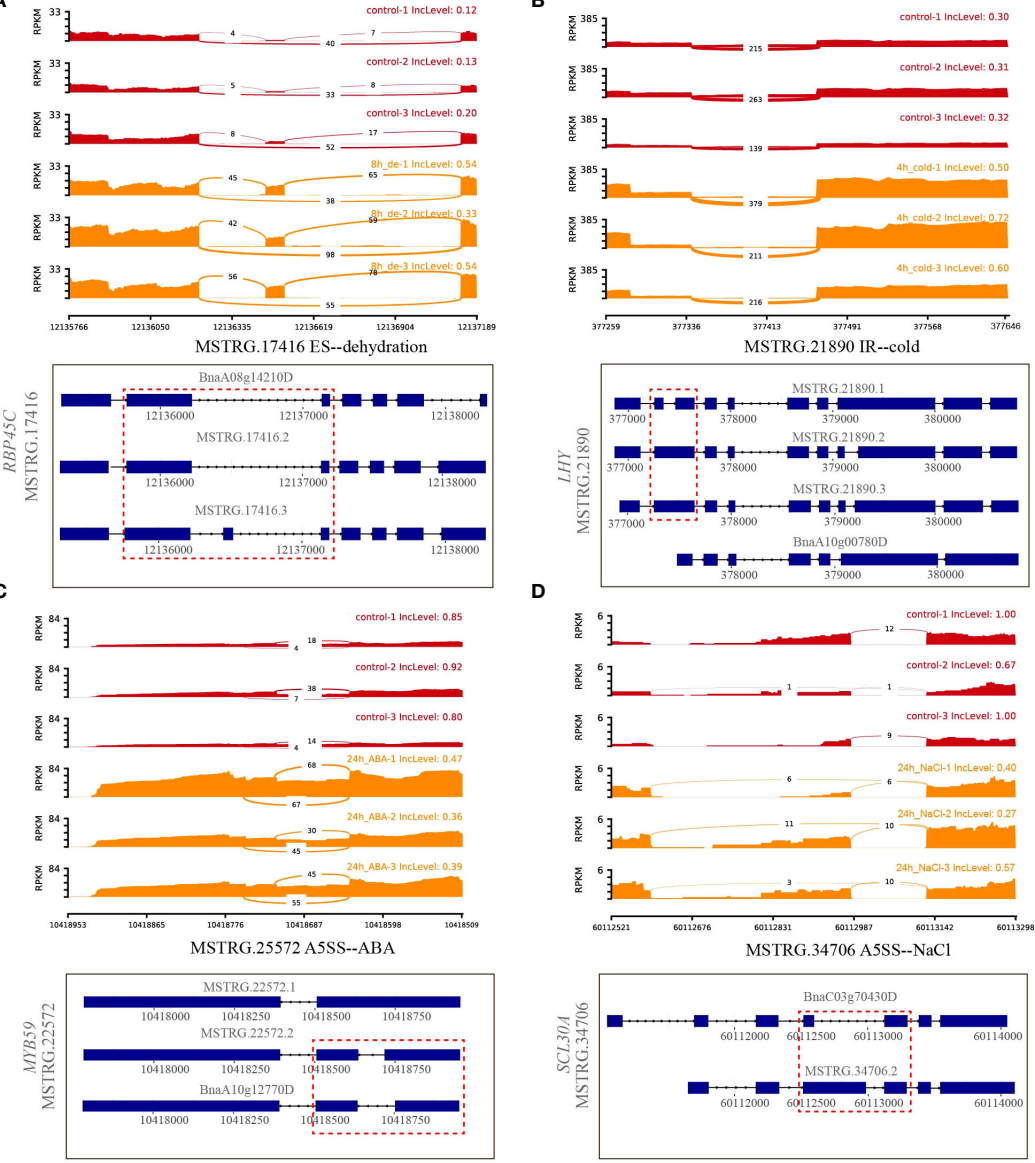
Quantitative visualization (sashimiplot) and transcripts structures of DAS genes under single abiotic stress. Panels **(A–D)** represented differential alternative splicing of different genes under dehydration, cold, ABA, and salt stress, respectively. Red dashed box indicated exons and introns showed in sashimiplots corresponding to gene structures below. The IncLevel value represented the normalized proportion of AS event. The red color denote control, and orange color denote treatments. The blue rectangular boxes denote the exons, the lines denote the introns, and the arrows denote the transcriptional direction.

A total of 32 genes producing hub isoforms within stress-related modules were differentially alternatively spliced only under dehydration stress, including those encoding RNA-binding (RRM/RBD/RNP motifs) family protein, glycine-rich protein, tyrosine kinase family protein, and RING/U-box superfamily protein (**Supplementary Table 5**). For example, *MSTRG.17416*, encoding an RRM motif containing protein, was DAS through ES events under dehydration for 8 h. Based on the structure analysis of gene transcripts, ES events were found to occur between *BnaA08g14210D*/*MSTRG.17416.2* and *MSTRG.17416.3* (**Figure 5A**). Notably, the IncLevels of *MSTRG.17416* were much higher under dehydration for 8 h than those of the mock control (**Figure 5A**), which indicated that this gene was prone to reduce ES events and retain exon 3 as much as possible, resulting into the generation of *MSTRG.17416.3*. As for the transcript levels of these divergent isoforms, *MSTRG.17416.3* had the highest expression at 8 h of dehydration, which belonged to the turquoise module closely related to 8 h of dehydration (**Figure 8**). The protein encoded by *MSTRG.17416.3* contained only two RRM_1 domains, while those encoded by other isoforms contained one more RRM_1 domain. Moreover, this gene was not differentially expressed, suggesting that alternative splicing provides an additional layer of transcriptional regulation in response to abiotic stress.

##### 3.3.1.2 Hub DAS genes under cold stress

In total, 56 hub DAS genes were found only under cold stress. These genes mainly encode homeodomain-like superfamily protein, WRKY DNA-binding protein 11, SC35-like splicing factor 33, DEAD/DEAH box RNA helicase, tyrosine kinase family protein, and RING/U-box superfamily protein (**Supplementary Table 5**). Among them, the MYB_related family TF *MSTRG.21890* (*LHY*) encoding a homeodomain-like superfamily protein is homologous to *AT1G01060*, which was involved in circadian rhythm along with another myb transcription factor *CCA1* in *Arabidopsis* (Adams et al., 2018). This gene experienced multiple AS events under cold stress for 4 h, including IR, ES, and A3SS, and also underwent IR event at 24 h of cold (**Supplementary Table 5**). Accordingly, IR events occurred between *MSTRG.21890.1* and *MSTRG.21890.2*/*MSTRG.21890.3* (**Figure 5B**). Remarkably, the IncLevels of IR events of *MSTRG.21890* were much higher than those of control (**Figure 5B**). In addition, the transcript level of *MSTRG.21890.1* was higher than that of the control at 4 h of cold (**Figure 8**). These results denoted that *MSTRG.21890* tended to produce more *MSTRG.21890.1* isoform that is responsive to cold stress.

##### 3.3.1.3 Hub DAS genes under ABA stress

Next, eight hub DAS genes were specifically found in response to ABA stress, including genes encoding cytochrome C1 family, cyclic nucleotide-binding transporter 1, and myb domain protein 59 *MSTRG.22572* (*MYB59*) (**Supplementary Table 5**). Remarkably, *AT4G31550*, the homolog of *MYB59* in *Arabidopsis*, has been reported to respond to ABA and cold stresses (Naika et al., 2013). This gene was identified as a DAS gene through A5SS event at 24 h of ABA (**Supplementary Table 5**), and the IncLevels were notably lower than the control, suggesting a tendency to increase the transcript of *BnaA10g12770D* (**Figure 5C**). Moreover, *BnaA10g12770D* displayed the highest expression level at 24 h of ABA, which was designated to the magenta module linked to 24 h of ABA (**Figure 8**). All of these findings indicated that *MSTRG.22572* was likely to generate *BnaA10g12770D* isoform in response to ABA stress.

##### 3.3.1.4 Hub DAS genes under NaCl stress

However, five hub DAS genes were identified only in NaCl stress, including *SCL30A*, SR30, *HD2B*, *SLP1*, and *MQB2.8* (**Supplementary Table 5**). One splicing factor *MSTRG.34706* (*SCL30A*) was differentially spliced *via* A5SS event at 24 h of NaCl (**Supplementary Table 5**). From the structures of gene transcripts, the A5SS event occurred between *BnaC03g70430D* and *MSTRG.34706.2* (**Figure 5D**). The IncLevels of *MSTRG.34706* were considerably lower than control at 24 h of NaCl stress (**Figure 5D**), suggesting that this gene enhanced the expression of *BnaC03g70430D*. Interestingly, this gene was not a differentially expressed gene (DEG), indicating that alternative splicing is of crucial importance for abiotic stress tolerance in *B. napus*.

#### 3.3.2 Hub DAS genes in response to multiple abiotic stresses

**Figure 6:**
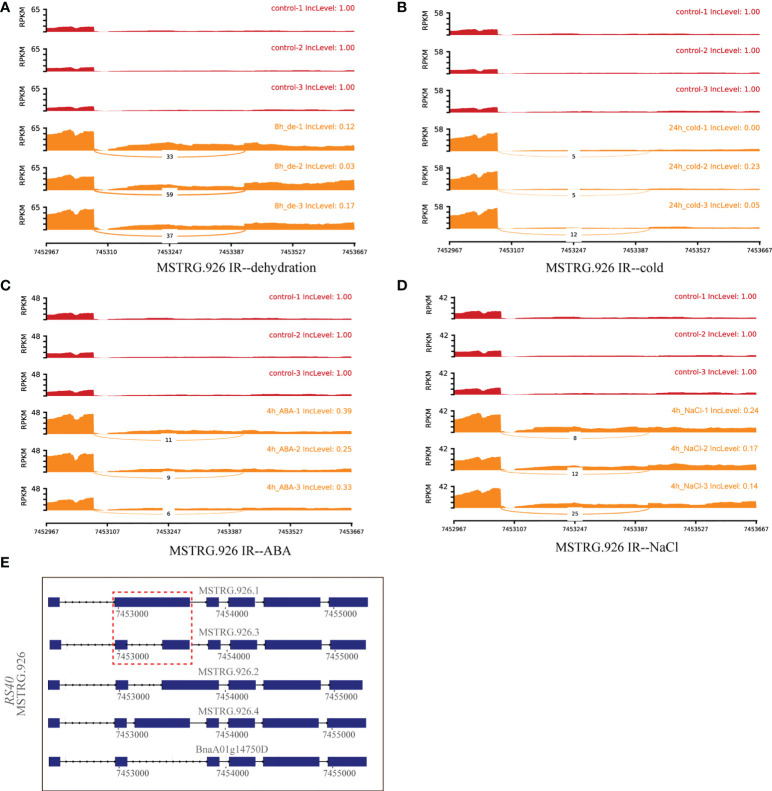
Quantitative visualization (sashimiplot) and transcripts structures of the DAS gene (*MSTRG.926*, RBP) under multiple abiotic stresses. Panels **(A–D)** represent dehydration, cold, ABA, and NaCl stress, respectively. The IncLevel value represents the normalized proportion of AS event. The red color denotes control, and orange color denotes treatments. Panel **(E)** displays the structures of all transcripts of this gene. Red dashed box indicates exons and introns shown in sashimiplots corresponding to gene structures below. The blue rectangular boxes denote the exons, the lines denote the introns, and the arrows denote the transcriptional direction.

A total of 30 DAS genes generating hub isoforms in stress-related modules were detected under at least two stresses. These genes mainly encode RRM/RBD/RNP motif protein, DEAD/DEAH box RNA helicase, ser-/arg-rich protein 34A, arginine-/serine-rich splicing factor 35, homeodomain-like superfamily protein, circadian clock associated 1, glycine-rich protein, cytochrome P450 protein, and RING/U-box superfamily protein (**Supplementary Table 5**). Among them, *MSTRG.926* (*RS40*), encoding arginine/serine-rich splicing factor 35, was differentially alternatively spliced through IR event under multiple stress treatments, such as dehydration (8 h), cold (24h), ABA (4 h), and NaCl (4 h) (**Figures 6A–D**). Structure analyses revealed that IR event occurred between *MSTRG.926.1* and *MSTRG.926.3* (**Figure 6E**), and all IncLevels of *MSTRG.926* showed much lower than those of the control under aforementioned treatments. *MSTRG.926.1* had the highest expression level at 8 h of dehydration, which existed in the turquoise module closely related to 8 h of dehydration (**Figure 8**). These findings implied that this splicing factor encoding gene showed the same AS pattern in response to different stress conditions. Furthermore, one RBP *MSTRG.1012*, encoding RRM/RBD/RNP motif protein, was also shown as DAS by ES event under several stress treatments (**Supplementary Figure 4**). However, *MSTRG.1012* was detected as a DEG only under dehydration and NaCl, but not ABA, which indicated that differential alternative splicing was partially independent of differential expression.

**Figure 7:**
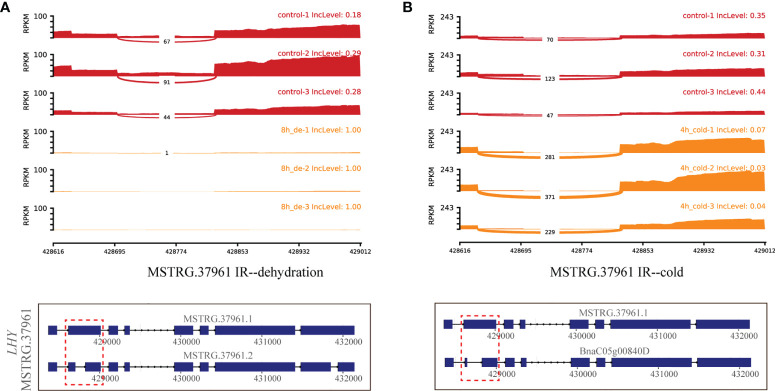
Quantitative visualization (sashimiplot) and transcripts structures of the DAS gene (*MSTRG.37961*, TF) under multiple abiotic stresses. Panels **(A, B)** represent dehydration and cold stress, respectively. Red dashed box indicates exons and introns shown in sashimiplots corresponding to gene structures below. The IncLevel value represents the normalized proportion of AS event. The red color denotes control, and orange color denotes treatments. The blue rectangular boxes denote the exons, the lines denote the introns, and the arrows denote the transcriptional direction.

**Figure 8:**
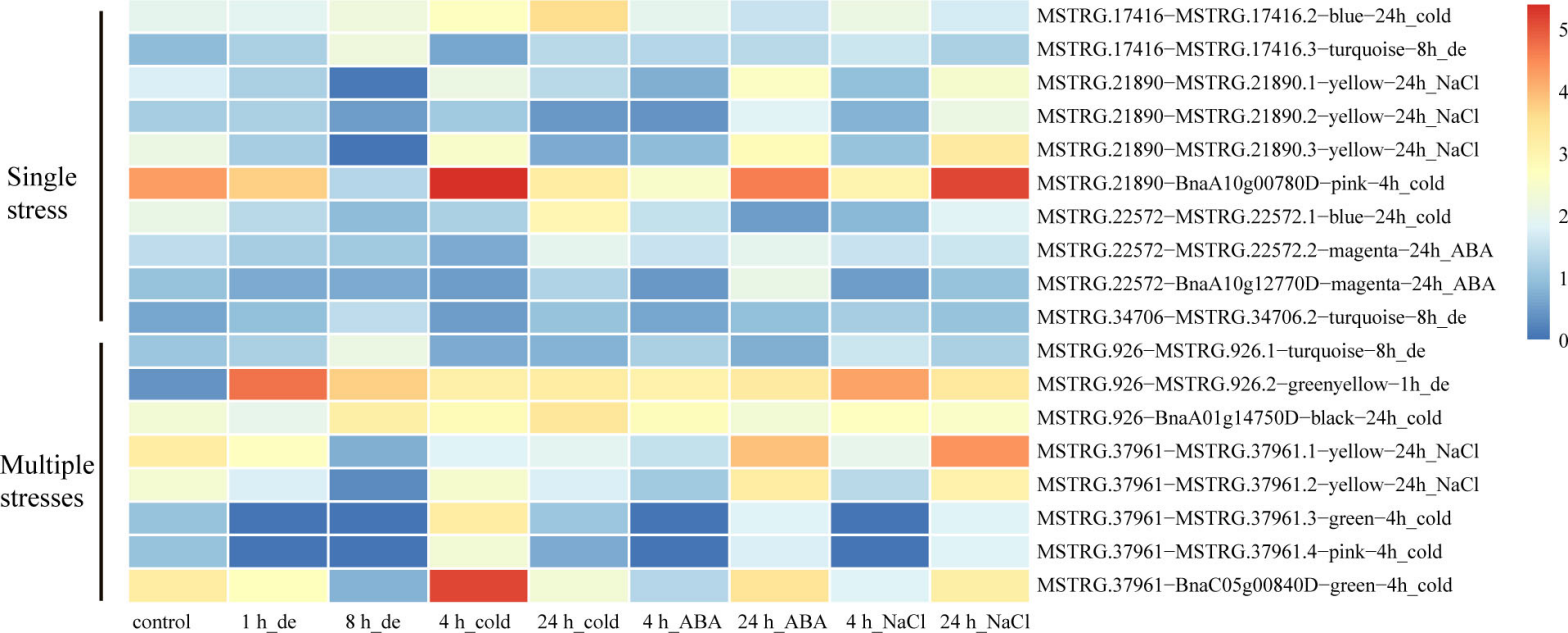
Expression heatmaps of each isoform of the candidate DAS genes under single stress or multiple stresses. The texts written behind the isoforms signifies their contained co-expression module that related to corresponding stress.

Additionally, the myb-related transcription factor *MSTRG.37961* (*LHY*), encoding homeodomain-like superfamily protein, experienced different IR events between *MSTRG.37961.1* and MSTRG.37961.2/BnaC05g00840D under dehydration for 8 h and cold stress for 4 h (**Figure 7**). Remarkably, the IncLevels of IR event of *MSTRG.37961* were significantly higher than what was found for the control under dehydration for 8 h (**Figure 7A**), whereas they were much lower under cold stress for 4 h (**Figure 7B**). Meanwhile, the transcript *BnaC05g00840D* displayed the highest expression at 4 h of cold, which was present in the green module associated with 4 h of cold (**Figure 8**). These results suggested that this TF encoding gene underwent different alternative splicing patterns in response to different stresses. Taken together, one gene may experience same or different AS patterns in response to diverse stresses.

## 4 Discussion

In this study, we aimed to determine the role of AS in response to multiple abiotic stresses by performing comparative transcriptome analyses on *B. napus* accession ZS11 at two different time points after treatments of dehydration, cold, ABA, and NaCl. Identification of DAS and analysis of co-expression networks at isoform level were conducted to elucidate plant internal response of AS to abiotic stresses. A total of 357 DAS genes were identified between control and treatments of the aforementioned four stresses, among which 131 DAS genes produced hub isoforms in the corresponding stress-related modules by WGCNA. These hub DAS genes included those encoding RNA-binding proteins, TFs, RING/U-box superfamily proteins, tyrosine kinase superfamily proteins, cytochrome P450 superfamily protein, and Transducin/WD40 repeat-like superfamily protein.

### 4.1 The potential relationship of AS and expression level in plants

In the original analysis of Zhang et al. (2019), a total of 30,908 DEGs were identified under these four abiotic stresses, which were involved in small molecules and cellular macromolecule metabolism, ribosome biogenesis, and translation (Zhang et al., 2019). These two different analyses originating from the same transcriptome data led us to consider whether there is a potential link between AS and expression level. The subsequent comparative analysis revealed that a total of nearly 53% DAS genes were differentially expressed under all stress conditions (**Supplementary Table 6**). With the time course of different treatments, the ratios of DEGs from DAS genes showed an increasing trend under dehydration and low temperature, ranging from 33% to 67% and from 35% to 61%, respectively, while it showed an opposite trend under NaCl treatment, with the ratio of 62% to 39% (**Supplementary Figure 5**). Moreover, the proportions of differentially expressed DAS genes were similar at two time points after ABA treatment, about 54% and 51%, respectively (**Supplementary Figure 5**). Remarkably, a previous study also pinpointed out that approximately 42% DAS genes were identified as DEGs under combined heat and drought stresses in wheat (Liu et al., 2018). These results suggested that AS can work together with expression regulation to contribute to abiotic stress responses. Nevertheless, some studies indicated that AS might function independently to gene expression under abiotic and biotic stresses (Gu et al., 2019; Ma et al., 2019; Ma et al., 2020; Li et al., 2022a). Thereafter, the exact relationship between AS and expression level in response to stress needs to be further investigated in detail.

### 4.2 The roles of RBPs in response to abiotic stress

RBPs play an important role in the post-transcriptional gene regulation, such as modulating the activity and fate of RNA transcripts by binding to the specific target RNAs (Marondedze, 2020). A multitude of RBP-encoding genes were found to be induced under various abiotic stress conditions and played a comprehensive role in the process of response (Yan et al., 2022). In this study, we identified 16 RBP-encoding genes, such as *CCR1*, *SR34a*, *SCL30A*, *SCL33*, *SR30*, *RS40*, *U1-70K*, *U1A*, and *U2AF65A*, by integrating the DAS genes and hub genes identified from co-expression modules. One *CCR1* gene *MSTRG.34246* (*GRP8*), encoding a glycine-rich protein with RNA binding domain, was identified to be a DAS gene in reaction to dehydration, ABA, and NaCl stresses. *AtGRP8* was previously reported to affect AS of downstream targets in *Arabidopsis* (Streitner et al., 2012). Furthermore, SR protein, a type of RBP, functions in pre-mRNA splicing and subsequent steps of post-transcriptional gene expression. It is composed of one or two RNA recognition motifs and a ser-/arg-rich domain responsible for recognizing pre-mRNA and recruiting spliceosomal components, respectively. It cannot only regulate the splicing of downstream target genes but also can be alternatively spliced itself (Joris et al., 2017; Xie et al., 2022). A growing body of evidence have reported that SR protein encoding genes underwent AS events in response to environmental stresses in plants. For example, *SR45a* and *RS2Z33* produced different isoforms to respond to drought stress in *G. uralensis* (Li et al., 2022b). A few *SR* genes were certificated to exhibit responsive AS patterns under heat, drought, and salt stress conditions in wheat (Liu et al., 2018). In *B. napus*, the AS modes of SR splicing factors have also been reported to change under different stress conditions, such as boron deficiency, *S. sclerotiorum*, and *L. maculans* (Gu et al., 2019; Ma et al., 2019; Ma et al., 2020). In this study, two *SR34a* (*MSTRG.46899* and *MSTRG.27677*) produced different isoforms in response to dehydration and cold stresses, suggesting that there was a certain responsive relationship between dehydration and cold. *RS40* (*MSTRG.926*) also experienced more IR events under four abiotic stresses, indicating that it was a common splicing factor responding to multiple stresses. *SCL30A* (*MSTRG.34706*) and *SR30* (*MSTRG.38321*) were differentially alternatively spliced under NaCl stress, while *SCL33* (*MSTRG.11046*) was a DAS gene under cold stress. These results indicated that RBP-encoding genes play an important role in response to various abiotic stresses *via* alternative splicing.

Moreover, *U1-70K*, a U1 snRNP-specific protein encoding gene, plays a crucial role in the early steps of spliceosome formation *via* binding to 5′ splice sites and is involved in basic and alternative splicing of nuclear pre-mRNAs. *U1-70K* is important in a lot of biological processes, such as the development of sepals and petals and stress resistance response in *Arabidopsis* (Golovkin and Reddy, 2003; Amorim et al., 2018). In this study, *U1-70K* (*MSTRG.48887*) was found to undergo DAS to produce different isoforms belonging to two distinct modules (turquoise and blue) related to 8 h of dehydration and 24 h of cold, respectively. It was reported that U1 snRNP protein can interact with serine-/arginine-rich proteins to regulate splicing of pre-mRNAs in *Arabidopsis* (Golovkin and Reddy, 2003). A similar phenomenon was also found in our work, according to the co-expression of *U1-70K* and *SR34a* in the turquoise and blue modules. Spliceosomal protein encoding gene *U1A* (*MSTRG.42942*) was also a DAS gene identified in this study under stresses of dehydration and NaCl. Notably, a previous study demonstrated that *U1A* was involved in pre-mRNA processing and salt tolerance in *A. thaliana* (Gu et al., 2018). In addition, we also found that U2 snRNP auxiliary factor encoding gene, *U2AF65A* (*MSTRG.17488*), was DAS by A3SS event under 4 h of cold. *OsU2AF65A* was certificated to mediate response to various stresses, such as drought, cold, high salt, and heavy metal exposure in rice (Lu et al., 2021).

### 4.3 The roles of TFs in response to abiotic stress

Transcription factors are proteins that bind to specific *cis*-acting response elements in the promoters of stress-inducible genes, which can modulate the transcription of target genes and control key downstream responses, thus enhancing the stress tolerance in plants. In this study, two *LHY* genes (*MSTRG.37961* and *MSTRG.21890*) and one *CCA1* gene (*MSTRG.9519*) were identified to be DAS genes in reaction to dehydration and/or cold stresses. *LHY* played a crucial role in promoting the expression of ABA-responsive genes, which improved *Arabidopsis* tolerance to drought and osmotic stresses (Yamaura et al., 2020). Furthermore, AS of *LHY* was also proved to influence circadian rhythm, and *CCA1* can produce two isoforms in *Arabidopsis*, a full-length isoform *CCA1α* and a truncated isoform *CCA1β* resulting from an intron retention, which exhibited enhancive tolerance and sensitivity to cold, respectively (Seo et al., 2012). AS of *CCA1* was also reported to regulate the circadian clock in response to low temperature in *B. napus* (Lee and Adams, 2020).

In our work, *HSFA1E* (*MSTRG.11411*) and *WRKY11* (*MSTRG.26624*) were differentially alternatively spliced owing to cold stress. The heat shock transcription factors, *HSFA1D* and *HSFA6B*, were induced to produce different isoforms by AS under drought and heat stresses in wheat (Liu et al., 2018). *WRKY19* and *WRKY20* were also prone to generate corresponding isoform under drought stress in *G. uralensis* (Li et al., 2022a). A DAS gene (*MSTRG.22572*) encoding myb domain protein 59 was identified in responding to ABA stress. *MYB59* was recently reported to respond to low K^+^ stress in *Arabidopsis* (Du et al., 2019), and its transcriptional level was also influenced by alternative splicing in *Arabidopsis* and rice (Li et al., 2006). These results revealed that transcription factors cannot only activate or inhibit gene transcription, but also undergo constitutive and inducible AS under abiotic stresses. Interestingly, we found that TFs tended to co-express with RBPs in the modules related to various abiotic stresses, and both of them were hub genes/isoforms, which was consistent with previous reports (He and Hu, 2021).

### 4.4 The roles of other DAS genes in response to abiotic stress

Several other important DAS genes were also pinpointed in response to abiotic stress. For example, RING/U-box superfamily protein encoding gene *MSTRG.50841* was DAS by A5SS event under cold, ABA, and NaCl stresses. It is homologous to *AT5G10650*, which was involved in ABA-mediated microtubule depolymerization and tolerance response to drought stress (Yu et al., 2020). A DAS gene *MSTRG.27581* (*DWF4*), encoding P450 protein, was identified in reaction to dehydration, cold, and NaCl stresses. *DWF4* played a key role in brassinosteroid (BR) homeostasis and was related to root elongation in BR-auxin crosstalk in *Arabidopsis* (Rahman et al., 2011). Transducin/WD40 repeat-like superfamily protein encoding gene *MSTRG.42815* was also a DAS gene identified in this study under stresses of dehydration and cold. Its homolog in *Arabidopsis*, *AT1G78070*, was involved in drought, cold, ABA, and NaCl stresses (Naika et al., 2013). In addition, *MSTRG.16072* and *MSTRG.42565*, encoding tyrosine kinase family proteins, were DAS genes under dehydration and cold stress, respectively. Both of these two genes were homologous to *AT1G73660*, which may function as a negative regulator of salt tolerance (Gao and Xiang, 2008).

## 5 Conclusions

In this study, a total of 357 DAS genes were identified by comparative transcriptome analyses under abiotic stress conditions, such as dehydration, cold, ABA, and salt stress. Through isoform-based WGCNA, 23 co-expression modules related to different stresses were determined, and a number of crucial genes, such as RBPs and TFs, changed AS modes in responding to single abiotic stress or multiple stresses in *B. napus*. The identified potential candidate isoforms could be useful targets for further functional studies to explore the molecular mechanism of AS in response to abiotic stresses. Taken together, this study provides novel insights of AS dynamics associated with abiotic stresses in *B. napus* and forms a beneficial resource for molecular breeding to create cultivars tolerant to multiple stresses not only in *B. napus* but also in other crops.

## Data availability statement

The datasets presented in this study can be found in online repositories. The names of the repository/repositories and accession number(s) can be found in the article/**Supplementary Material**.

## Author contributions

LLY, MX, and SL designed the research. LLY performed the research. JL, CZ, YZ, CT and XC supplied assistance in data analysis. LLY wrote the draft manuscript. MX, LY, HJ, and JS revised the manuscript. CT and SL applied the funding. All authors contributed to the article and approved the submitted version.

## Funding

This research was funded by the Yong Top-notch Talent Cultivation Program of Hubei Province for Dr. Chaobo Tong, the National Natural Science Foundation of China (No. 31770250), the Agricultural Science and Technology Innovation Program of Chinese Academy of Agricultural Sciences (CAAS-ASTIP-2013-OCRI), and the Earmarked Fund for China Agriculture Research System (CARS-12).

## Acknowledgments

We sincerely thank Prof. Liang Guo (Huazhong Agricultural University) for publishing and uploading the sequencing data to the public database. We sincerely thank Zhixian Qiao of the Analysis and Testing Center at IHB for technical supports in RNA-seq analysis.

## Conflict of interest

The authors declare that the research was conducted in the absence of any commercial or financial relationships that could be construed as a potential conflict of interest.

## Publisher’s note

All claims expressed in this article are solely those of the authors and do not necessarily represent those of their affiliated organizations, or those of the publisher, the editors and the reviewers. Any product that may be evaluated in this article, or claim that may be made by its manufacturer, is not guaranteed or endorsed by the publisher.

## Supplementary material

Supplementary Figure 1 IGV showing the RNA-seq reads aligned to each transcript of DAS gene under control and stress conditions. **(A, B)** represented MSTRG.1012 and MSTRG.17416, respectively. Green box indicated the position of the skipped exons and the reads spanning the junction. The transcripts structures were presented as dark blue exon-plots at the bottom: boxes represented the exons, lines represented the introns, and arrows represented the transcriptional direction.

Supplementary Figure 2 Distribution of the number of genes/isoforms contained in each module.

Supplementary Figure 3 Top20 GO terms enrichment of genes in the seven stress-related modules. **(A-G)** respectively represented brown, turquoise, green, pink, blue, magenta, and yellow module. The x-axis represented the -log10(Pvalue) which shows the significant level. The size of the bubble represented the enriched gene number.

Supplementary Figure 4 Quantitative visualization (sashimiplot) and transcripts structures of the DAS gene (MSTRG.1012, RBP) under multiple abiotic stresses. **(A-C)** represented dehydration, ABA and NaCl stress, respectively. The IncLevel value represented the normalized proportion of AS event. The red color denoted control and orange color denoted treatments. **(D)** displayed the structures of all transcripts of this gene. Red dashed box indicated exons and introns showed in sashimiplots corresponding to gene structures below. The blue rectangular boxes denoted the exons, the lines denoted the introns, and the arrows denoted the transcriptional direction.

Supplementary Figure 5 The percentage of DAS genes overlapping with DEGs under each stress condition.
