# Supplementary figures and images for: Differential alternative splicing genes and isoform co-expression networks of Brassica napus under multiple abiotic stresses
Source: Front Plant Sci. 2022 Oct 13;13:1009998. doi: 10.3389/fpls.2022.1009998 (PMC9608124; doi:10.3389/fpls.2022.1009998)

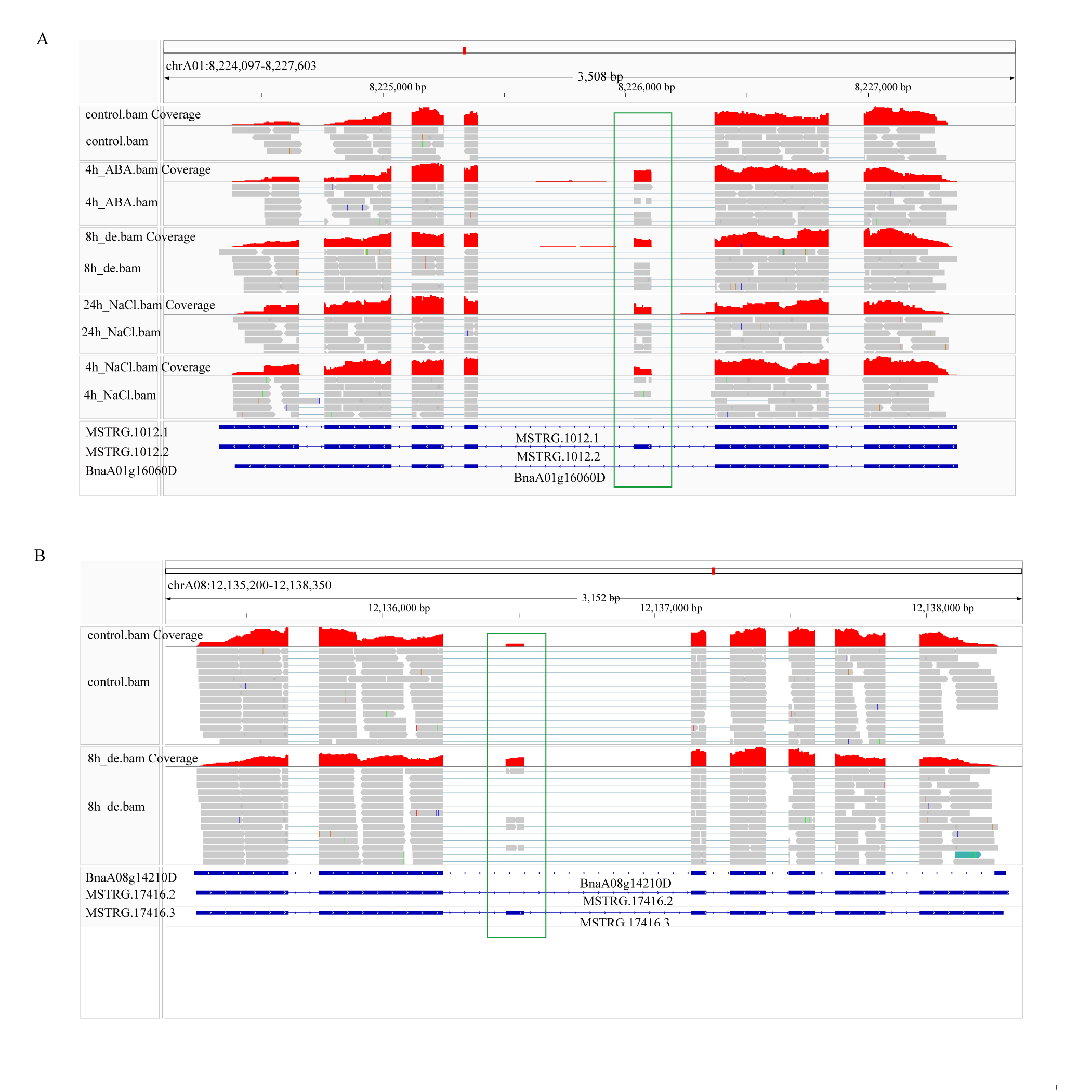

Supplement: Supplementary Figure 1 — IGV showing the RNA-seq reads aligned to each transcript of DAS gene under control and stress conditions. (A, B) represented MSTRG.1012 and MSTRG.17416, respectively. Green box indicated the position of the skipped exons and the reads spanning the junction. The transcripts structures were presented as dark blue exon-plots at the bottom: boxes represented the exons, lines represented the introns, and arrows represented the transcriptional direction. [file Image_1.tif]

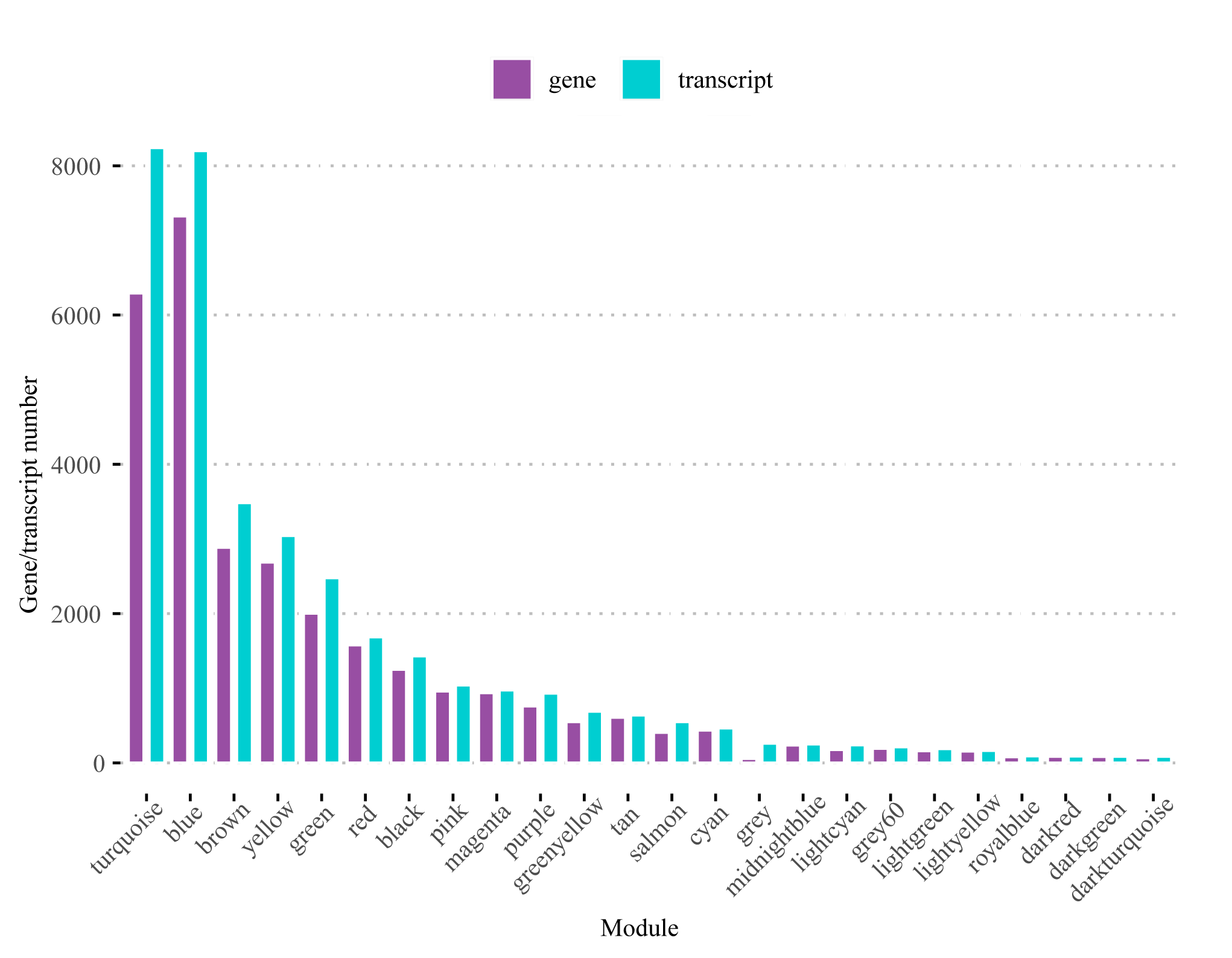

Supplement: Supplementary Figure 2 — Distribution of the number of genes/isoforms contained in each module. [file Image_2.tif]

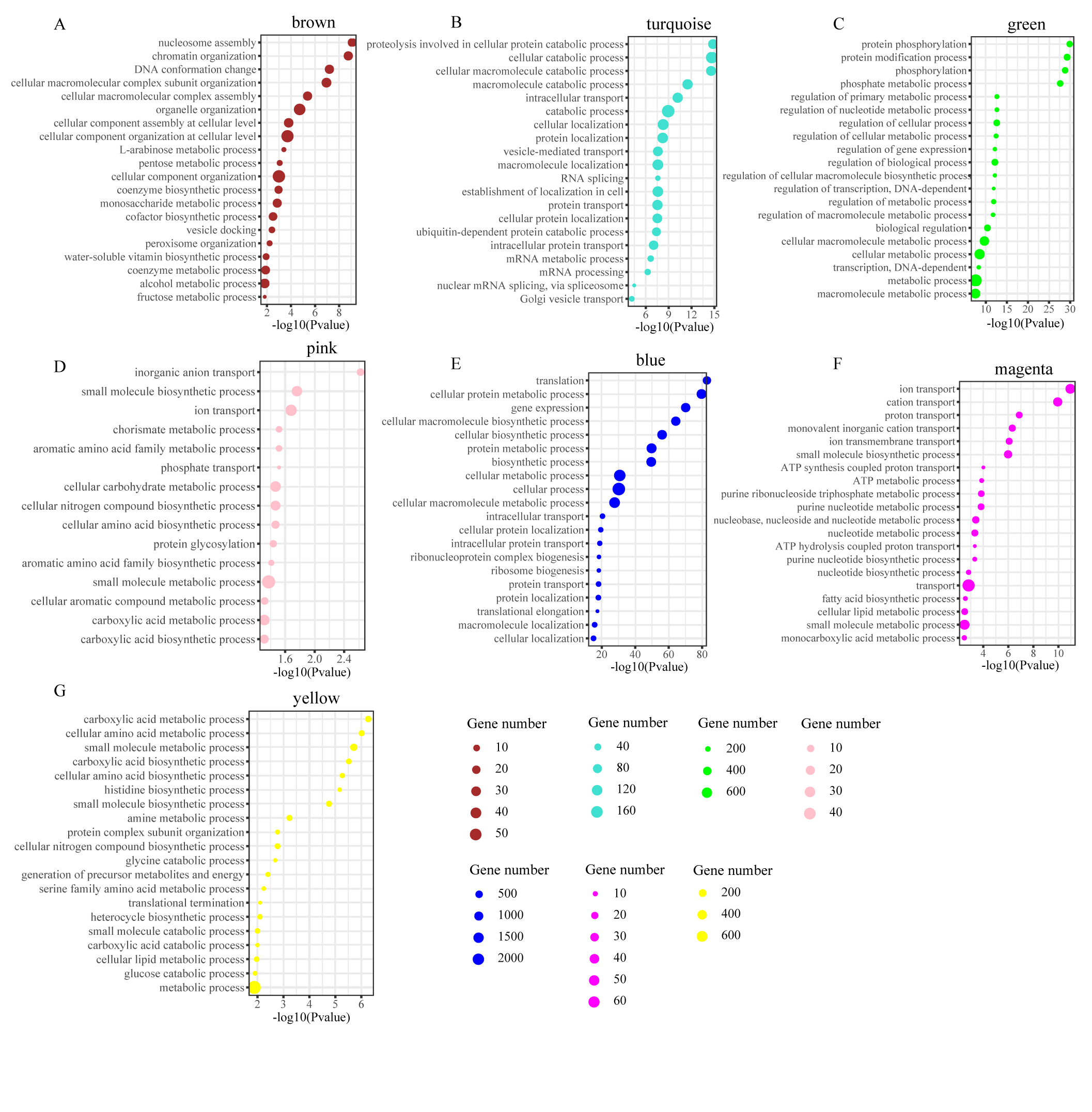

Supplement: Supplementary Figure 3 — Top20 GO terms enrichment of genes in the seven stress-related modules. (A-G) respectively represented brown, turquoise, green, pink, blue, magenta, and yellow module. The x-axis represented the -log10(Pvalue) which shows the significant level. The size of the bubble represented the enriched gene number. [file Image_3.tif]

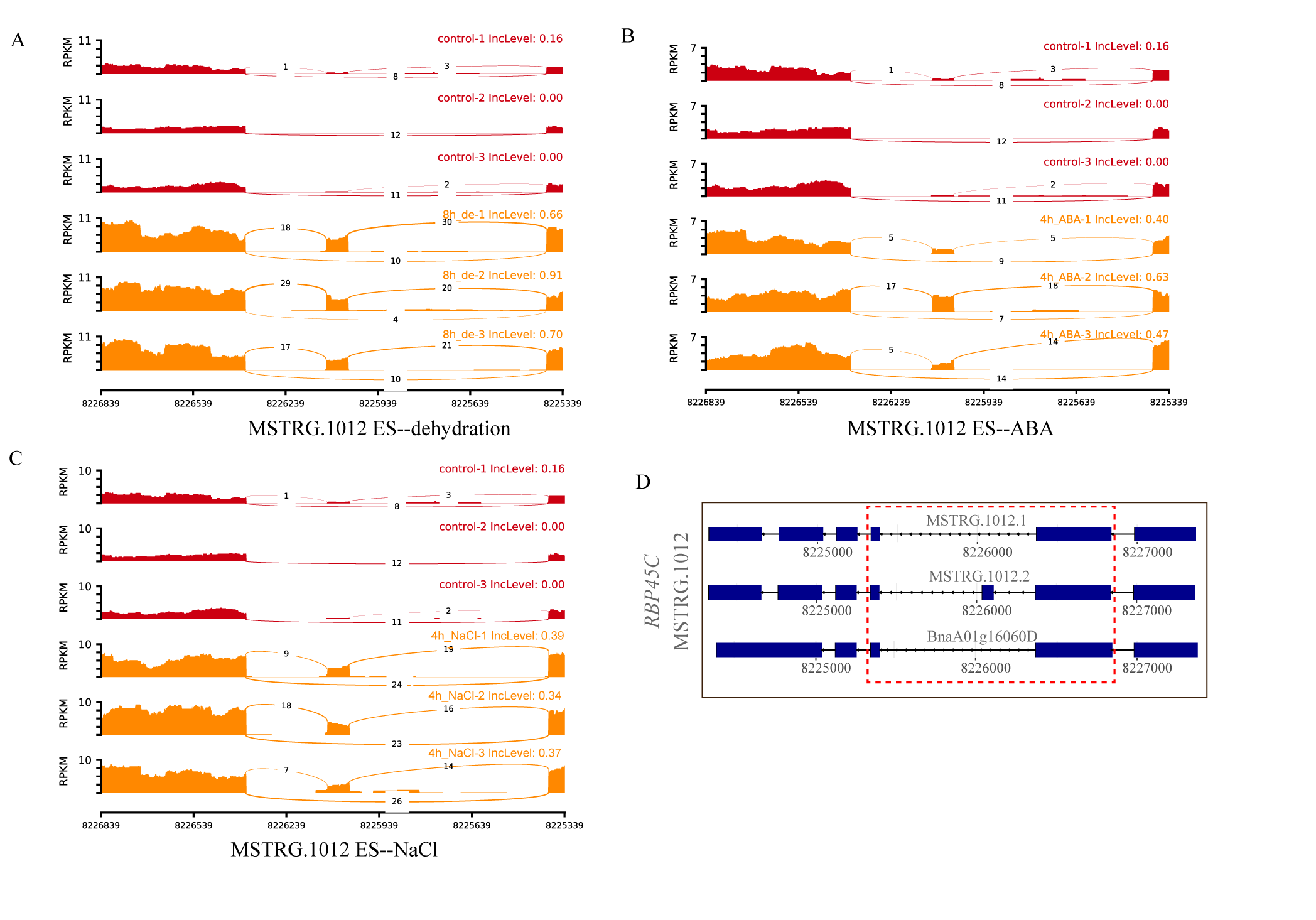

Supplement: Supplementary Figure 4 — Quantitative visualization (sashimiplot) and transcripts structures of the DAS gene (MSTRG.1012, RBP) under multiple abiotic stresses. (A-C) represented dehydration, ABA and NaCl stress, respectively. The IncLevel value represented the normalized proportion of AS event. The red color denoted control and orange color denoted treatments. (D) displayed the structures of all transcripts of this gene. Red dashed box indicated exons and introns showed in sashimiplots corresponding to gene structures below. The blue rectangular boxes denoted the exons, the lines denoted the introns, and the arrows denoted the transcriptional direction. [file Image_4.tif]

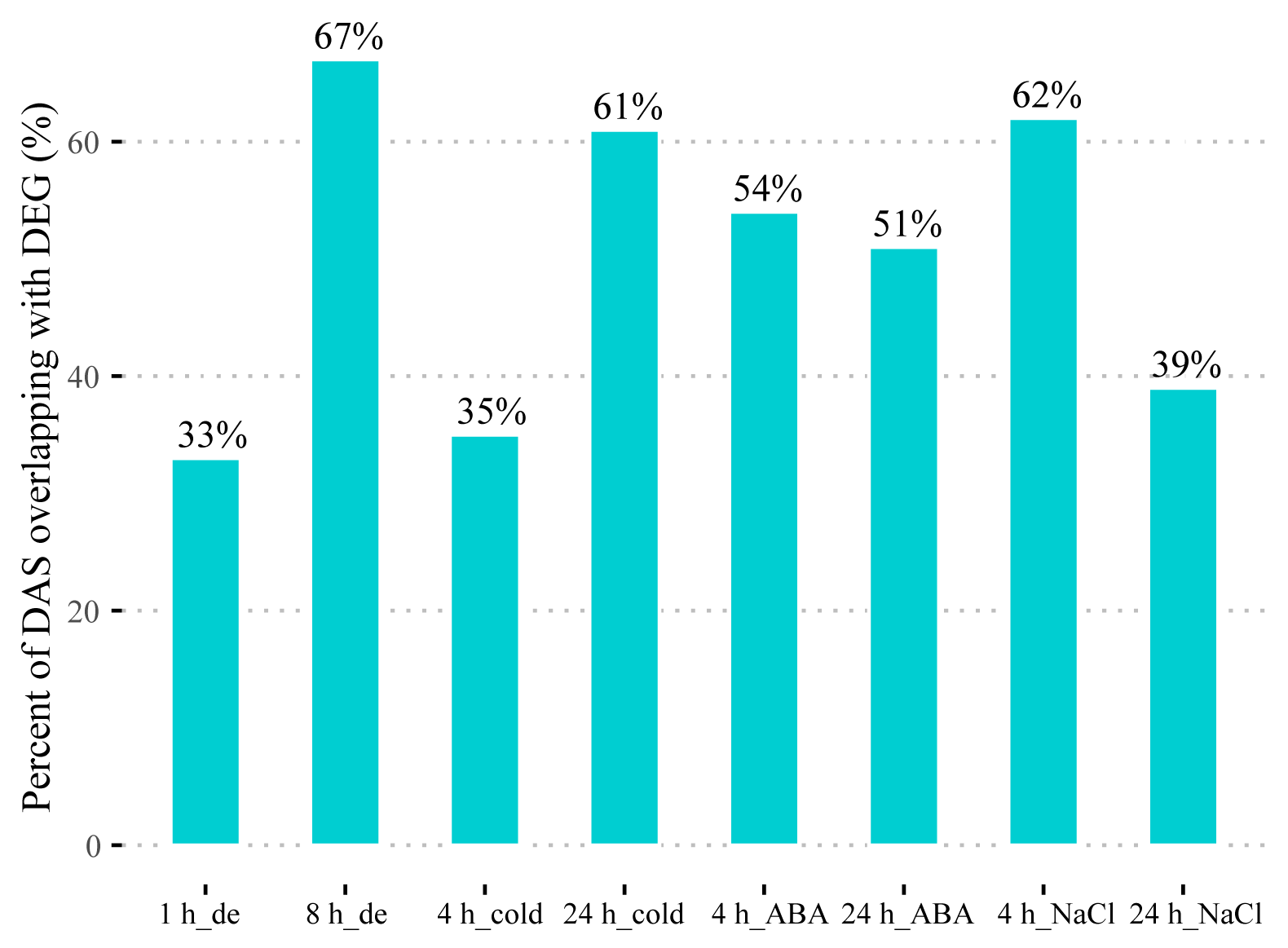

Supplement: Supplementary Figure 5 — The percentage of DAS genes overlapping with DEGs under each stress condition. [file Image_5.tif]

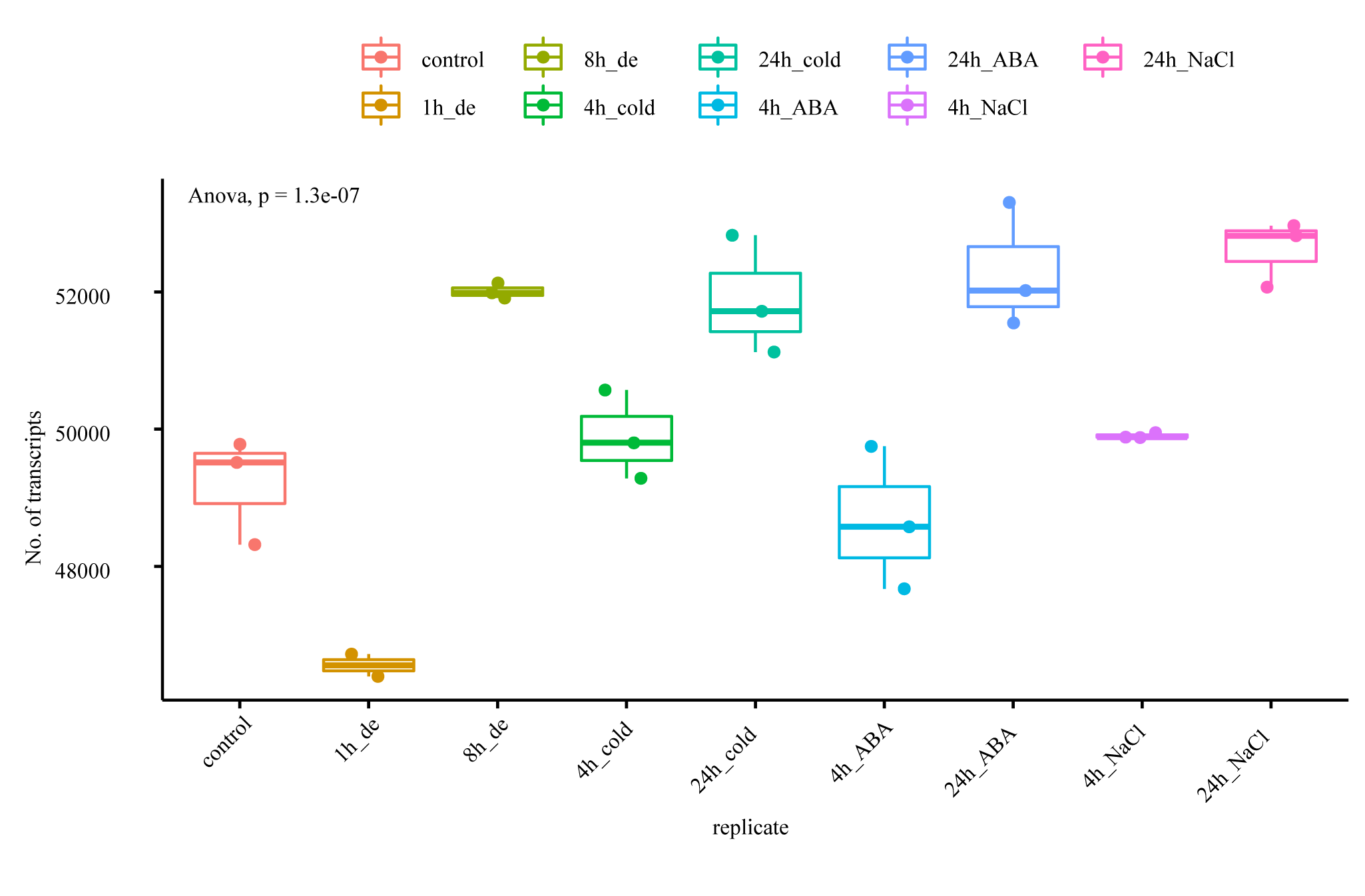

Supplement: Supplementary file 6 [file Image_6.tif]
